# Some Provings of Merc-Iod. CM, and Kali-Iod. 2d

**Published:** 1890-10

**Authors:** Ella M. Tuttle

**Affiliations:** New Berlin, New York


### SOME PROVINGS OF MERC-IOD., CM, AND KALI-IOD., 2D (X TRIT.).

Ella M. Tuttle, M. D., New Berlin, New York.

*Mind.*—Depressed, anxious about trifles.

*Head.*—A dull pain over the right eye. A sore aching pain over the right mastoid region extending to the ear, worse by pressure, by stooping, by jar, by thinking of it, better in the open air.

*Eyes.*—Lachrymation of the right eye, discharge bland, watery.

*Mouth.*—Smarting of the tongue. Dull pain in canine teeth on the right side.

*Throat.*—Burn in the left side of the throat extending to the stomach. Short, hacking cough caused by a feeling of irritation in the right side of the throat.

*Stomach.*—Nausea and cramping pains in the pit of the stomach.

*Extremities.*—Tired, aching pain on the outside of the left arm and forearm, extending to the fingers, followed by soreness and a feeling as if cold air were blowing on the arm, worse out-of-doors. Soreness of the anterior aspect of the thigh, as if it had been pounded. Tired feeling in the thigh.

